# Correction: Persistent high mortality rates for Diabetes Mellitus and Hypertension after excluding deaths associated with COVID-19 in Brazil, 2020–2022

**DOI:** 10.1371/journal.pgph.0004857

**Published:** 2025-06-25

**Authors:** Rodrigo Moreira, Leonardo S. Bastos, Luiz Max Carvalho, Laís Picinini Freitas, Antonio G. Pacheco

The ORCID iD of the second author is missing. Please see the authors’ ORCID iD here:

Author Leonardo S. Bastos’s ORCID iD is: 0000-0002-1406-0122 (https://orcid.org/0000-0002-1406-0122).
