# Supplementary figures and images for: Persistent high mortality rates for Diabetes Mellitus and Hypertension after excluding deaths associated with COVID-19 in Brazil, 2020–2022
Source: PLOS Glob Public Health. 2024 May 9;4(5):e0002576. doi: 10.1371/journal.pgph.0002576 (PMC11081286; doi:10.1371/journal.pgph.0002576)

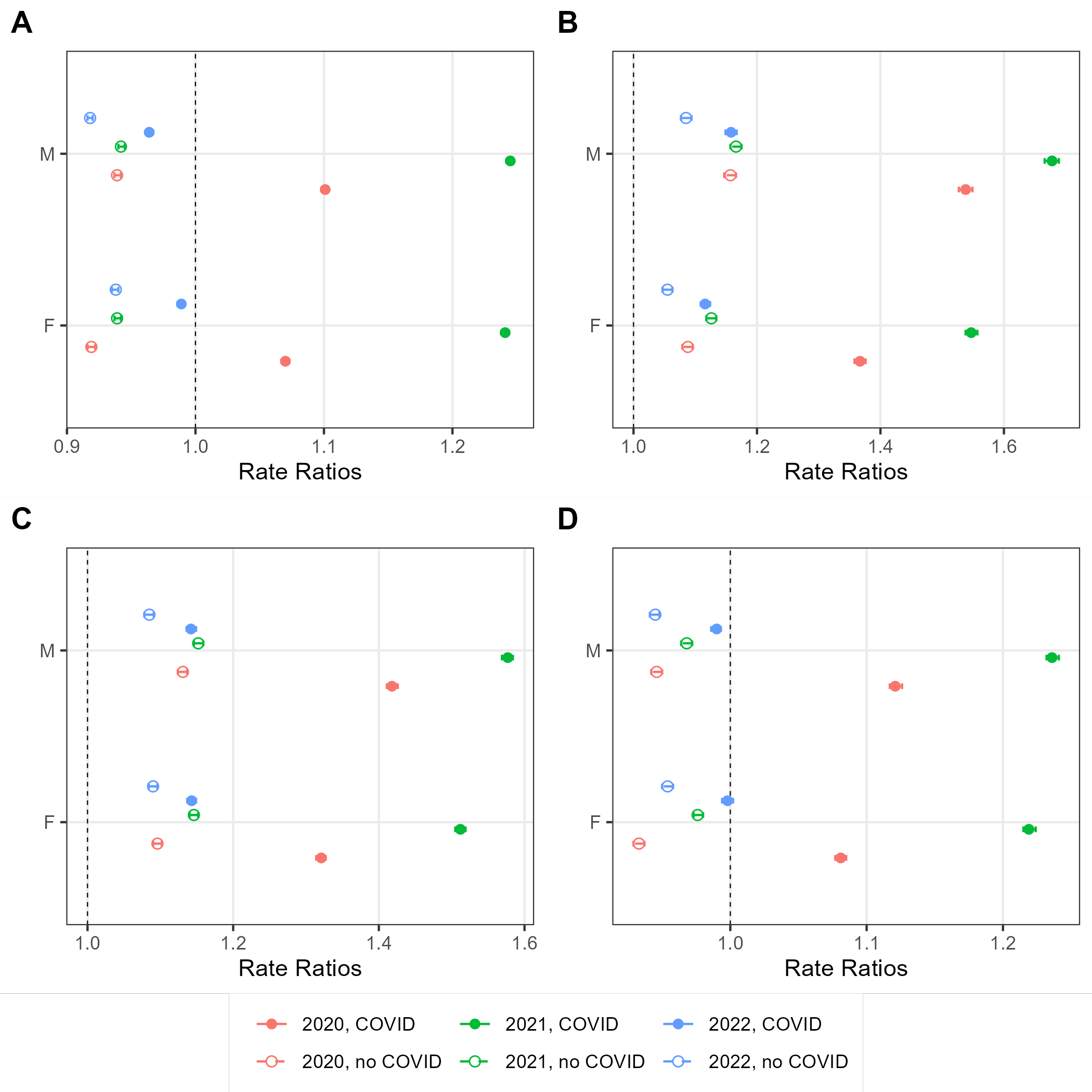

Supplement: S1 Fig — Adjusted Mortality Ratios in Brazil with and without COVID-19 mentioned in the death certificate per age group–A—Overall, B–DM, C–HTN, D–CVD; baseline—2015–2019). (TIFF) [file pgph.0002576.s001.tiff]

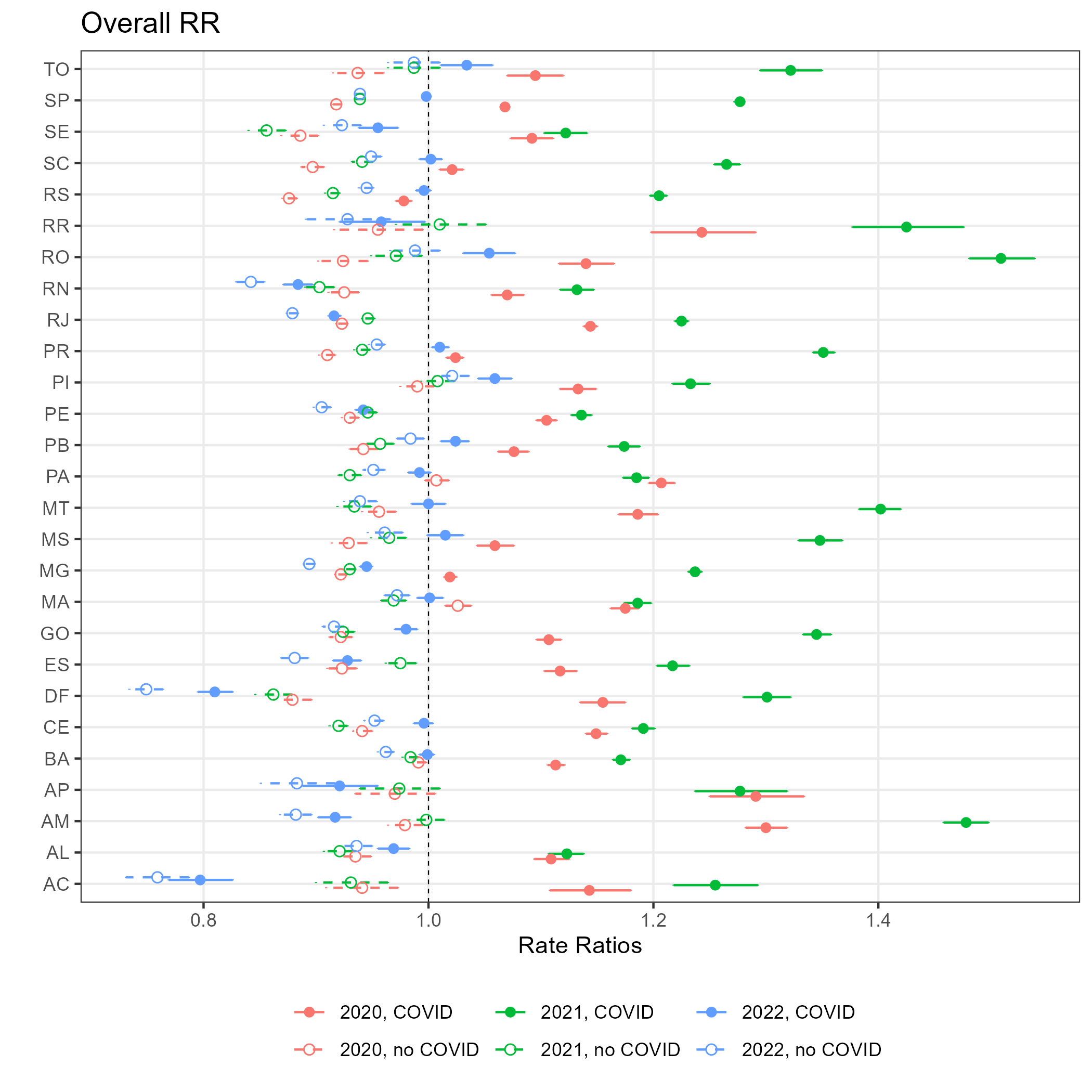

Supplement: S2 Fig — (TIFF) [file pgph.0002576.s002.tiff]

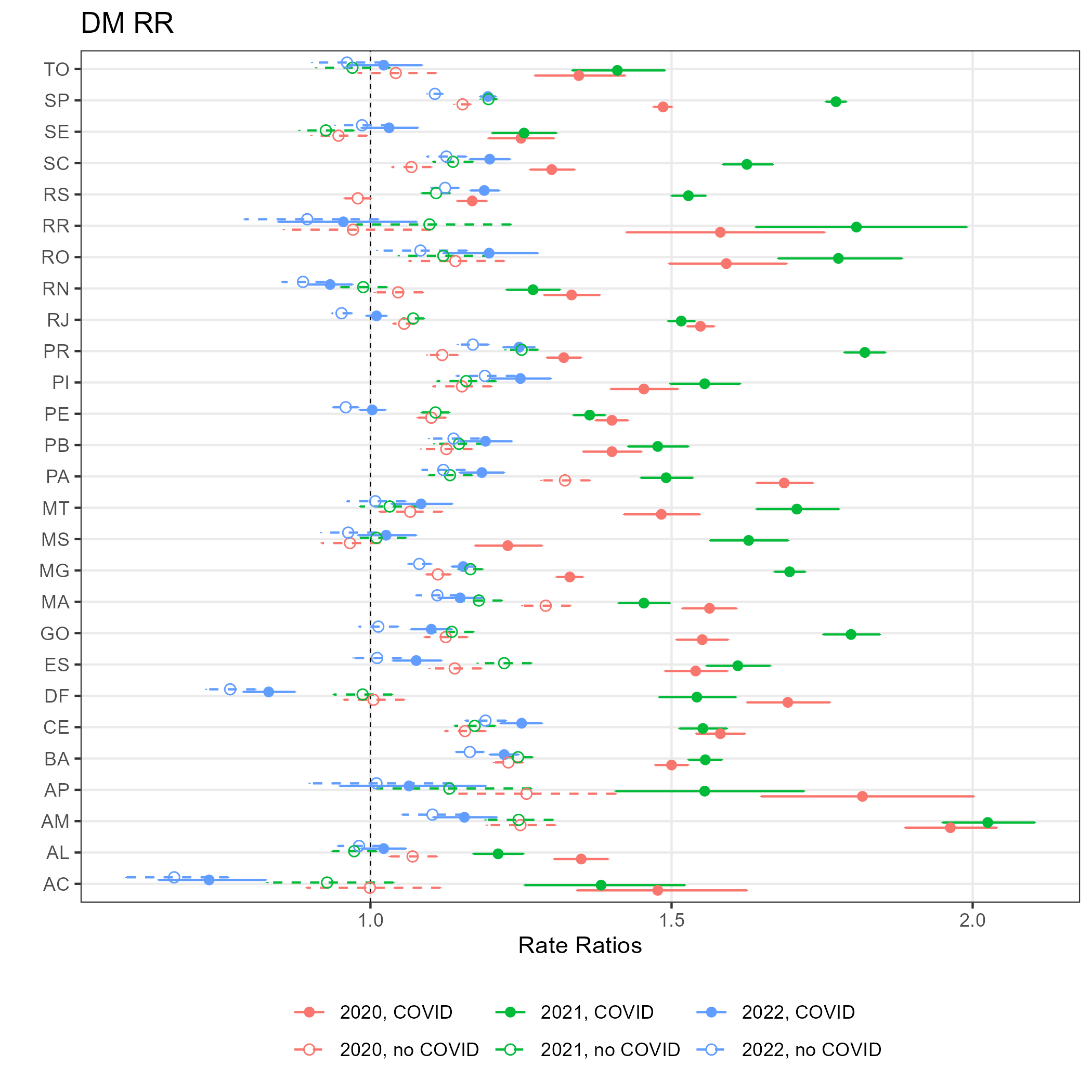

Supplement: S3 Fig — (TIFF) [file pgph.0002576.s003.tiff]

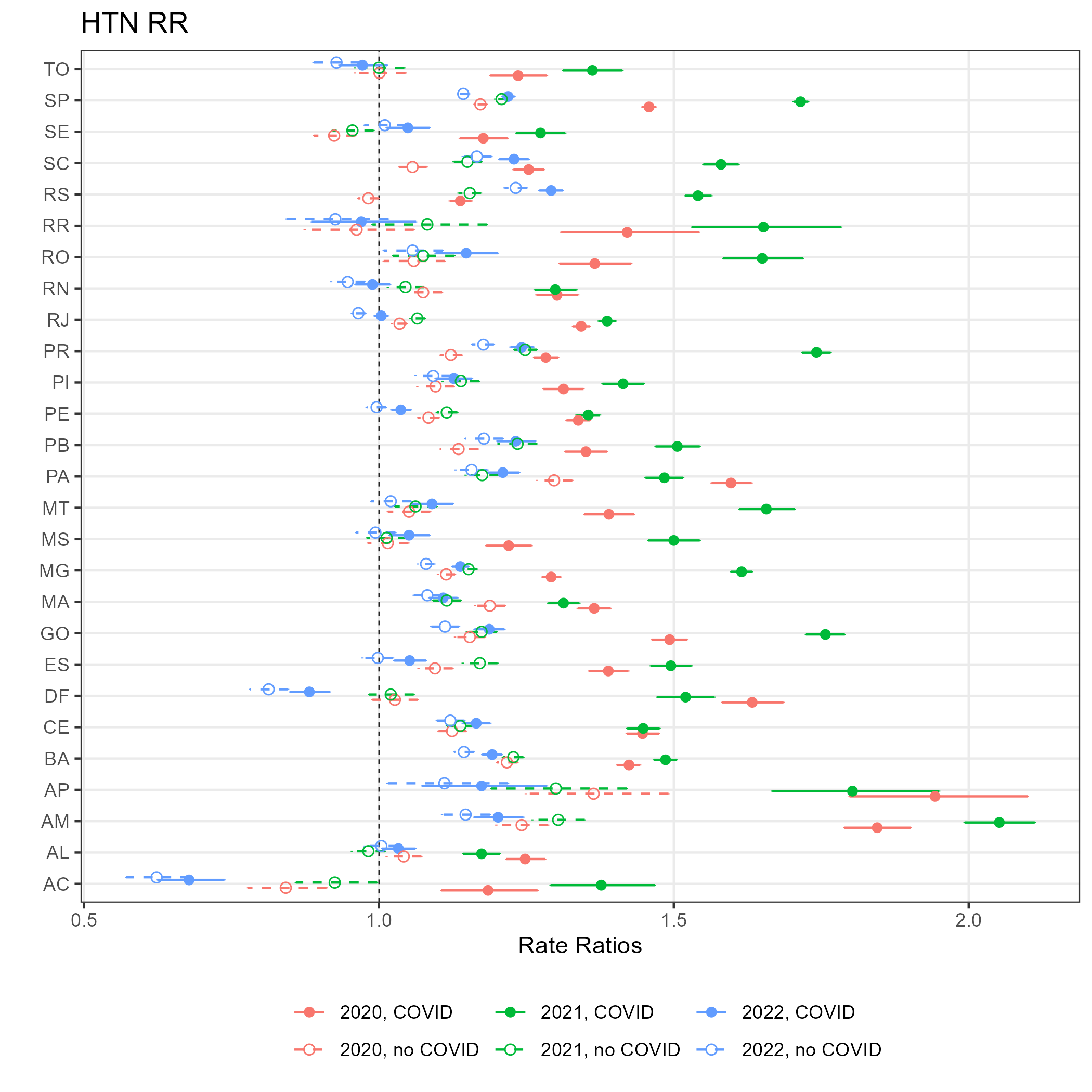

Supplement: S4 Fig — (TIFF) [file pgph.0002576.s004.tiff]

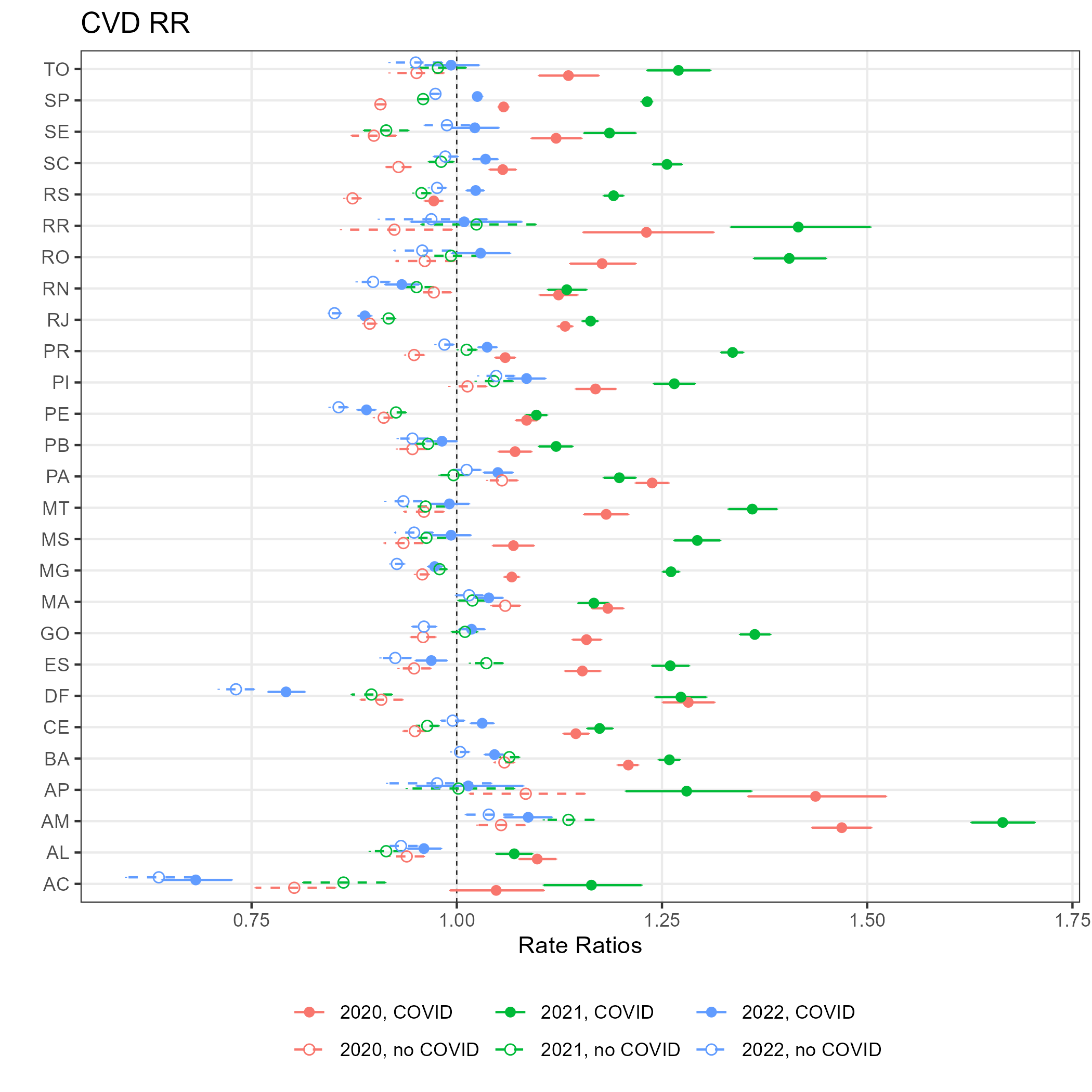

Supplement: S5 Fig — (TIFF) [file pgph.0002576.s005.tiff]
