# Supplementary material for: Persistent high mortality rates for Diabetes Mellitus and Hypertension after excluding deaths associated with COVID-19 in Brazil, 2020–2022
Source: PLOS Glob Public Health. 2024 May 9;4(5):e0002576. doi: 10.1371/journal.pgph.0002576 (PMC11081286; doi:10.1371/journal.pgph.0002576)
Supplement: S1 Table — (DOCX) [file pgph.0002576.s006.docx]

**Supplementary Table 1 - Characteristics of mortality data in Brazil, 2015-2022**

| Variables | 2015 | 2016 | 2017 | 2018 | 2019 | 2020 | 2021 | 2022 | Test | P-value |
| --- | --- | --- | --- | --- | --- | --- | --- | --- | --- | --- |
| *Total* | 1264175 | 1309774 | 1312663 | 1316719 | 1349801 | 1556824 | 1832649 | 1480683 |  |  |
| **Sex** |  |  |  |  |  |  |  |  | Chisq. (7 df) = 1315.92 | < 0.001 |
| *F* | 554383 (43.9) | 572359 (43.7) | 577573 (44) | 582457 (44.3) | 603725 (44.7) | 682027 (43.8) | 816616 (44.6) | 671639 (45.4) |  |  |
| *M* | 709117 (56.1) | 736842 (56.3) | 734469 (56) | 733616 (55.7) | 745519 (55.3) | 874167 (56.2) | 1015350 (55.4) | 808431 (54.6) |  |  |
| **Age group** |  |  |  |  |  |  |  |  | Chisq. (49 df) = 49139.97 | < 0.001 |
| *0-19* | 72611 (5.8) | 72524 (5.6) | 71517 (5.5) | 68270 (5.2) | 65206 (4.8) | 59129 (3.8) | 59026 (3.2) | 57736 (3.9) |  |  |
| *20-29* | 54336 (4.3) | 55643 (4.3) | 55755 (4.3) | 51952 (4) | 48862 (3.6) | 52953 (3.4) | 56836 (3.1) | 48890 (3.3) |  |  |
| *30-39* | 64190 (5.1) | 64864 (5) | 63776 (4.9) | 61151 (4.7) | 59984 (4.5) | 68164 (4.4) | 85202 (4.7) | 60468 (4.1) |  |  |
| *40-49* | 90993 (7.2) | 92650 (7.1) | 89177 (6.8) | 88826 (6.8) | 88957 (6.6) | 106488 (6.9) | 143975 (7.9) | 96536 (6.5) |  |  |
| *50-59* | 152331 (12.1) | 157797 (12.1) | 153293 (11.7) | 154401 (11.8) | 155434 (11.5) | 184102 (11.8) | 240690 (13.2) | 160319 (10.8) |  |  |
| *60-69* | 209620 (16.6) | 221752 (17) | 223284 (17) | 229156 (17.4) | 236822 (17.6) | 286190 (18.4) | 350131 (19.1) | 258228 (17.5) |  |  |
| *70-79* | 256180 (20.3) | 265220 (20.3) | 267012 (20.4) | 271919 (20.7) | 282040 (20.9) | 334733 (21.5) | 390064 (21.3) | 320458 (21.7) |  |  |
| *80+* | 360634 (28.6) | 376145 (28.8) | 385882 (29.5) | 388271 (29.5) | 410138 (30.4) | 462678 (29.8) | 504348 (27.6) | 475925 (32.2) |  |  |
| **DM** |  |  |  |  |  |  |  |  | Chisq. (7 df) = 27237.77 | < 0.001 |
| *No* | 1142083 (90.3) | 1181838 (90.2) | 1179632 (89.9) | 1178859 (89.5) | 1206536 (89.4) | 1343735 (86.3) | 1587388 (86.6) | 1301339 (87.9) |  |  |
| *Yes* | 122092 (9.7) | 127936 (9.8) | 133031 (10.1) | 137860 (10.5) | 143265 (10.6) | 213089 (13.7) | 245261 (13.4) | 179344 (12.1) |  |  |
| **HBP** |  |  |  |  |  |  |  |  | Chisq. (7 df) = 41441.79 | < 0.001 |
| *No* | 1038326 (82.1) | 1071858 (81.8) | 1063293 (81) | 1061823 (80.6) | 1085979 (80.5) | 1182248 (75.9) | 1394843 (76.1) | 1144904 (77.3) |  |  |
| *Yes* | 225849 (17.9) | 237916 (18.2) | 249370 (19) | 254896 (19.4) | 263822 (19.5) | 374576 (24.1) | 437806 (23.9) | 335779 (22.7) |  |  |
| **CVD** |  |  |  |  |  |  |  |  | Chisq. (7 df) = 2234.39 | < 0.001 |
| *No* | 740173 (58.5) | 764716 (58.4) | 764823 (58.3) | 762319 (57.9) | 781090 (57.9) | 887565 (57) | 1060667 (57.9) | 834580 (56.4) |  |  |
| *Yes* | 524002 (41.5) | 545058 (41.6) | 547840 (41.7) | 554400 (42.1) | 568711 (42.1) | 669259 (43) | 771982 (42.1) | 646103 (43.6) |  |  |
| **State of residence** |  |  |  |  |  |  |  |  | Chisq. (182 df) = 15510.2 | < 0.001 |
| *AC* | 3517 (0.3) | 3763 (0.3) | 3832 (0.3) | 4094 (0.3) | 4098 (0.3) | 4860 (0.3) | 5496 (0.3) | 3607 (0.2) |  |  |
| *AL* | 19756 (1.6) | 20769 (1.6) | 20673 (1.6) | 19411 (1.5) | 20287 (1.5) | 24148 (1.6) | 25090 (1.4) | 22238 (1.5) |  |  |
| *AM* | 16675 (1.3) | 16799 (1.3) | 17281 (1.3) | 17710 (1.3) | 18327 (1.4) | 24765 (1.6) | 29080 (1.6) | 18696 (1.3) |  |  |
| *AP* | 2946 (0.2) | 2995 (0.2) | 3158 (0.2) | 3345 (0.3) | 3524 (0.3) | 4617 (0.3) | 4750 (0.3) | 3565 (0.2) |  |  |
| *BA* | 87083 (6.9) | 88094 (6.7) | 90915 (6.9) | 90134 (6.8) | 93365 (6.9) | 107194 (6.9) | 115392 (6.3) | 100629 (6.8) |  |  |
| *CE* | 55258 (4.4) | 54276 (4.1) | 59263 (4.5) | 57028 (4.3) | 56580 (4.2) | 69512 (4.5) | 73683 (4) | 62942 (4.3) |  |  |
| *DF* | 11975 (0.9) | 12050 (0.9) | 12514 (1) | 12157 (0.9) | 12804 (0.9) | 16218 (1) | 19079 (1) | 12433 (0.8) |  |  |
| *ES* | 22332 (1.8) | 22868 (1.7) | 24112 (1.8) | 23500 (1.8) | 24431 (1.8) | 29111 (1.9) | 32801 (1.8) | 25818 (1.7) |  |  |
| *GO* | 38854 (3.1) | 38074 (2.9) | 39973 (3) | 39507 (3) | 41025 (3) | 48358 (3.1) | 60712 (3.3) | 45731 (3.1) |  |  |
| *MA* | 33666 (2.7) | 34362 (2.6) | 35275 (2.7) | 34525 (2.6) | 35128 (2.6) | 43271 (2.8) | 44654 (2.4) | 38510 (2.6) |  |  |
| *MG* | 131274 (10.4) | 135257 (10.3) | 138118 (10.5) | 135619 (10.3) | 141022 (10.4) | 152128 (9.8) | 190085 (10.4) | 149449 (10.1) |  |  |
| *MS* | 15457 (1.2) | 16749 (1.3) | 15954 (1.2) | 16600 (1.3) | 16815 (1.2) | 19051 (1.2) | 25049 (1.4) | 19476 (1.3) |  |  |
| *MT* | 17095 (1.4) | 17535 (1.3) | 17709 (1.3) | 18205 (1.4) | 18341 (1.4) | 23397 (1.5) | 28623 (1.6) | 21136 (1.4) |  |  |
| *PA* | 37365 (3) | 38557 (2.9) | 39980 (3) | 40513 (3.1) | 40599 (3) | 51643 (3.3) | 52166 (2.8) | 45017 (3) |  |  |
| *PB* | 26422 (2.1) | 28041 (2.1) | 26975 (2.1) | 26644 (2) | 27378 (2) | 31107 (2) | 34678 (1.9) | 30888 (2.1) |  |  |
| *PE* | 62556 (4.9) | 66928 (5.1) | 64364 (4.9) | 62011 (4.7) | 64295 (4.8) | 76574 (4.9) | 80717 (4.4) | 68818 (4.6) |  |  |
| *PI* | 19366 (1.5) | 19187 (1.5) | 19850 (1.5) | 19983 (1.5) | 20528 (1.5) | 23646 (1.5) | 26212 (1.4) | 22906 (1.5) |  |  |
| *PR* | 70839 (5.6) | 74740 (5.7) | 71633 (5.5) | 73848 (5.6) | 74566 (5.5) | 82573 (5.3) | 112606 (6.1) | 86930 (5.9) |  |  |
| *RJ* | 132714 (10.5) | 141089 (10.8) | 136709 (10.4) | 140706 (10.7) | 144600 (10.7) | 172185 (11.1) | 189232 (10.3) | 145337 (9.8) |  |  |
| *RN* | 20153 (1.6) | 21922 (1.7) | 21409 (1.6) | 21209 (1.6) | 21767 (1.6) | 24674 (1.6) | 26771 (1.5) | 21451 (1.4) |  |  |
| *RO* | 7948 (0.6) | 8344 (0.6) | 8219 (0.6) | 8165 (0.6) | 8338 (0.6) | 10278 (0.7) | 14042 (0.8) | 10130 (0.7) |  |  |
| *RR* | 2091 (0.2) | 2157 (0.2) | 2461 (0.2) | 2787 (0.2) | 2779 (0.2) | 3580 (0.2) | 4306 (0.2) | 3020 (0.2) |  |  |
| *RS* | 82349 (6.5) | 87583 (6.7) | 86241 (6.6) | 88618 (6.7) | 89238 (6.6) | 92791 (6) | 117722 (6.4) | 100142 (6.8) |  |  |
| *SC* | 37984 (3) | 40270 (3.1) | 39919 (3) | 41268 (3.1) | 42282 (3.1) | 46444 (3) | 59898 (3.3) | 49350 (3.3) |  |  |
| *SE* | 13453 (1.1) | 13516 (1) | 13321 (1) | 13024 (1) | 13473 (1) | 15793 (1) | 16675 (0.9) | 14605 (1) |  |  |
| *SP* | 287645 (22.8) | 296359 (22.6) | 294753 (22.5) | 298313 (22.7) | 306190 (22.7) | 349635 (22.5) | 431616 (23.6) | 348591 (23.5) |  |  |
| *TO* | 7402 (0.6) | 7490 (0.6) | 8052 (0.6) | 7795 (0.6) | 8021 (0.6) | 9271 (0.6) | 11514 (0.6) | 9268 (0.6) |  |  |
