# Supplementary material for: Persistent high mortality rates for Diabetes Mellitus and Hypertension after excluding deaths associated with COVID-19 in Brazil, 2020–2022
Source: PLOS Glob Public Health. 2024 May 9;4(5):e0002576. doi: 10.1371/journal.pgph.0002576 (PMC11081286; doi:10.1371/journal.pgph.0002576)
Supplement: Attachment — Submitted filename: response reviwers 29_2.docx [file pgph.0002576.s007.docx]

PGPH-D-23-02038

Persistent high mortality rates for Diabetes Mellitus and Hypertension after excluding deaths associated with COVID-19 in Brazil, 2020-2022 PLOS Global Public Health

Dear Dr. Steve Zimmerman,

Thank you for considering our manuscript entitled “Persistent high mortality rates for Diabetes Mellitus and Hypertension after excluding deaths associated with COVID-19 in Brazil, 2020-2022.” for publication in PLOS Global Public Health. We are submitting a revised version that takes into account the comments received following our initial submission.

A comprehensive point-by-point response to the Editor’s and reviewers’ comments is provided below. We would like to take the opportunity to once again thank the editor and the reviewers for their careful review and comments, that helped to improve our manuscript considerably.

Please contact me if you have any additional questions or comments about our manuscript.

Sincerely,

[Rodrigo]

**Journal Requirements:**

1-Please provide separate figure files in .tif or .eps format only and remove any figures embedded in your manuscript file. Please also ensure all files are under our size limit of 10MB. For more information about figure files please see our guidelines:https://journals.plos.org/globalpublichealth/s/figures
[https://journals.plos.org/globalpublichealth/s/figures#loc-file-requirement](about:blank)

Response: We have generated separate figure files in .tif formats. Additionally, we have removed all figures that were embedded in the manuscript file.

2. In the online submission form, you indicated that "Data will be available upon request". All PLOS journals now require all data underlying the findings described in their manuscript to be freely available to other researchers, either 1. In a public repository, 2. Within the manuscript itself, or 3. Uploaded as supplementary information.
This policy applies to all data except where public deposition would breach compliance with the protocol approved by your research ethics board. If your data cannot be made publicly available for ethical or legal reasons (e.g., public availability would compromise patient privacy), please explain your reasons by return email and your exemption request will be escalated to the editor for approval. Your exemption request will be handled independently and will not hold up the peer review process, but will need to be resolved should your manuscript be accepted for publication. One of the Editorial team will then be in touch if there are any issues.

Response: We appreciate the clarification on the PLOS data sharing policy. We selected the wrong option in our initial response, and we apologize for this confusion. In fact, all data used in our study come from secondary and publicly available data sources. We have included a statement with all the links to each dataset we used. The mortality data used in our study is sourced from the Brazilian mortality system (SIM - *Sistema de Informação sobre Mortalidade*), which is publicly available from DATASUS (Opendatasus - [https://opendatasus.saude.gov.br/dataset/sim](about:blank)). Additionally, population data were obtained from the Brazilian Census Bureau (IBGE – *Instituto Brasileiro de Geografia e Estatística*) through the SIDRA system ([http://api.sidra.ibge.gov.br/](about:blank)).

To comply with the PLOS data sharing policy, we ensure that the relevant data files are made publicly accessible through the provided links. This will enable other researchers to access and validate our findings.

**Review Comments to the Author**

**Reviewer #1:**

1-The present population-based study has scientific merit because analyzed mortality data from Brazil's mortality system, covering the period from 2015 to 2022. I believe that the results are interesting, but perhaps they can be applied to the local epidemiological reality. I have made some recommendations. Case detection using ICD-10 may be biased. How did the authors correct these possible biases?

Response: Thank you for the time you dedicated to reviewing our work and for your comments. In the Discussion section (lines 249-255 in the revised version), we acknowledge the inherent challenges associated with data derived from death certificates, a critical aspect raised by the reviewer. We address this concern by providing a transparent discussion on the limitations posed by potential misclassification in the death certificates, despite thorough revision before data entry into the database (1). We highlight the dual challenge of underreporting certain conditions, particularly when obscure or "garbage" ICD-10 codes are reported as the underlying cause of death, as well as the risk of inaccurate diagnoses. To tackle these challenges, our study employs a methodological approach that involves searching for all causes mentioned on death certificates. This strategy is implemented to mitigate the issues of underreporting and misclassification, and it has been successfully employed in previous research focused on studying causes of death in Brazil (2). We emphasize the rationale behind this approach, emphasizing its utility in capturing a more comprehensive representation of the causes of mortality.

2- How were the outcomes measured? Perhaps ICHOM is suitable for this study.

Response: The outcomes in our research were measured using publicly available information from individual death certificate data obtained from the Brazilian mortality system, managed by DataSUS, and population data from the Brazilian Census Bureau (IBGE). In response to your suggestion regarding ICHOM (International Consortium for Health Outcomes Measurement), we appreciate the recommendation. While ICHOM standards are valuable for standardizing and improving outcome measurements, our study relies on existing public health databases in Brazil, which does not allow direct adherence to ICHOM standards. However, these databases provide comprehensive information at a population level, allowing us to analyze trends and patterns over time. Due to the nature of our study, while ICHOM standards may be suitable for certain clinical studies, the population health focus of our research aligns with the available public health databases in Brazil.

3-Regarding continuous variables, have their distributions been evaluated? The Kolmogorov-Smirnov test with Lilliefors correction was applied?

Response: All variables used in the models were categorized. This is shown in the Supplementary Table 1.

4- there are few variables presented, I suggest showing more sociodemographic and clinical data of the sample evaluated.

Response: Thank you for your suggestion. We appreciate the importance of a comprehensive understanding of the study sample. However, we would like to provide context regarding the data limitations. The data utilized in our study were obtained from SIM (*Sistema de Informação sobre Mortalidade*). Unfortunately, the available data through this system were limited to certain variables, including location, age and sex. As a result, we were constrained by the available information and could not incorporate a broader set of sociodemographic and clinical variables. The current study reflects the available national database to address specific research questions within the scope of the provided variables.

5-In some situations, in table 1, the 95% CI has the same values for the lower and upper limits. How is this possible?

Response: Thank you for asking for clarification regarding the confidence intervals in Table 1. We understand that due to the large sample size and rounding to two decimal places, the calculated confidence intervals may have resulted in the same values as the point estimates so we changed Table 1 to tree decimal places.

6-A survival curve using the Kaplan-Meyer method could have been performed.

Response: In our study, we opted to calculate adjusted mortality ratios using Poisson models with the log of the population as an offset. This approach was chosen based on the nature of our data and research objectives, which focused on estimating mortality ratios while adjusting for potential confounding variables. Poisson regression models are commonly used for analyzing count and rate data, such as mortality rates, and allow for the inclusion of covariates to estimate adjusted mortality ratios. By incorporating the log of the population as an offset, we accounted for variations in population size across different time periods.

7- Calculate the statistical power of the evaluated sample.

Response: Given that our study utilized a secondary database with universal coverage, and not a sample, it did not involve a sample size calculation or power analysis.(SIM - *Sistema de Informação sobre Mortalidade*) The evaluated data are comprised of all deaths registered in Brazil in the study period. To make this clear, we have provided additional context regarding the size of the study population and the precision of our estimates in the Results, line 139 in the revised version.

8- Are cost variables available? The case-mix could be presented and added to the multivariate model. Clinical variables related to oxygen therapy could be shown and perhaps added to the multivariate model.

Response: Please see our response to your comment #4. The data utilized in our study were obtained from SIM. The available data through this system is limited to certain variables, including location, age, and sex. Regrettably, cost variables and clinical variables related to oxygen therapy were not available. As a result, we were unable to incorporate these variables into our analysis or multivariate model.

9- What are the statistical criteria used to use Poisson regression?

Response: Poisson regression is a statistical method used when the outcome variable of interest represents counts of events or rates. It is particularly suitable when the outcome variable is a non-negative integer such as number of deaths and follows a Poisson distribution, which is characterized by a mean equal to its variance. In this study deaths were recorded in Brazil from 2015 to 2022 and fitted in a Poisson regression model.

**Reviewer #2:**

I read with appreciation the interesting PGPH-D-23-02038 manuscript intitled “Persistent high mortality rates for Diabetes Mellitus and Hypertension after excluding deaths associated with COVID-19 in Brazil, 2020-2022” and some questions emerged.

1-To a full paper the introduction section is very short and discuss modestly the diseases evaluated and its epidemiological role in terms morbidity and mortality impact worldwide and in Brazil, before and during pandemic period. In other words, the study rationale seems absent and also there is no a hypothesis or something like.

Response: Thank you for dedicating time to review our work and for your feedback. We agree with the reviewer that it is important to ensure that the introduction section of a paper provides a comprehensive overview of the study rationale and hypothesis. We have made a thorough revision of the introduction and now it includes more background on COVID-19 morbidity and mortality impact worldwide and in Brazil in lines 58-78.

2- The sentence “even though the role of HTN and DM as independent risk factors are not yet clear (8)” needs a robust support, specifically regarding DM.2

Response: When addressing your comment #1, we have expanded the introduction with literature on the epidemiological gaps about HTN and DM in the context of COVID-19 (lines 69 - 73).

3- The excerpt "In this study we compared sex, age and state of residence adjusted mortality ratios (aMR) in Brazil in 2020-2022, compared to the preceding period of 2015-2019 and in subgroups of CVD, DM and HTN whenever these conditions were mentioned on death certificates. Comparisons were made with and without COVID-19 mentioned on the death certificates.", seems more appropriate into methods section, because the authors explain analytical procedures instead to state the overall manuscript purpose, for example. The third paragraph, could be better exploited, highlighting the CVD, HTM and DM rates or data in Brazil apart from a brief message to the reader on the indirect pandemic effects, including those taken as residual.

Response: Thank you for your feedback. Here's a revised version of the relevant sections:

“Methods:

In this study we compared sex, age and state of residence adjusted mortality ratios (aMR) in Brazil in 2020-2022, compared to the preceding period of 2015-2019 and in subgroups of CVD, DM and HTN whenever these conditions were mentioned on death certificates. Comparisons were made with and without COVID-19 mentioned on the death certificates."

In addition, we expanded the third paragraph, lines 64-68, to highlighting mortality rates in Brazil.

4- Please, to update the dataset source because the link https://opendatasus.saude.gov.br/dataset/sim is broken.

Response: Thank you for bringing this to our attention. The dataset source link provided ([https://opendatasus.saude.gov.br/dataset/sim](about:blank)) may have been temporarily offline. We checked from different locations and the link worked in all of them.

5-This is the latest version available to the 2022 year? There were exclusion criteria applied?
Please, to clarify what is the reason for differences between values to the total deaths added in the present analysis (11,423,288) regarding the total deaths available in “TabNet/Datasus” from Brazilian Ministry of Health (11,484,763 deaths).

Response: The 2022 database we used was deemed as preliminary data when downloaded for our study (on August/30/2023). This is acknowledged in the Methods section, line 100. The differences in total deaths between the data we used (11,423,288 deaths) and the total deaths available in "TabNet/Datasus" from the Brazilian Ministry of Health (11,484,763 deaths) are possibly because the latter is more up-to-date. We also would like to clarify that we did not employ any exclusion criteria.

6-The excerpt “All data from death certificates except information that could identify individuals were available for analysis…” needs more attention since the Brazilian Ministry of Health does not offer “All data from death certificates” but rather a simplified digital version, including variables solely administrative.

Response: Thank you for highlighting this important distinction. We have improved this sentence in lines 109-10, that now reads “All data from SIM consisted of variables extracted from a digital version of death certificates except information that could identify individuals and were available for analysis, including all causes of death”. We hope this addresses your concern.

7-Regarding sentence “…we worked with ICD-10 codes of interest mentioned in any field from the death certificates” the authors have worked with ICD-10 codes in “any field” or specific fields such as 49 field (section VI)?

Response: We appreciate this comment – the reviewer is right, we meant all fields where ICD-10 codes are expected to be found, i.e. fields from section VI-49. We have changed the text (lines 112-113), which now reads ‘we worked with ICD-10 codes of interest mentioned in any field where those codes are expected to be found (i.e. section VI-49)’.

7-The Figure 1 might be improved through the transformation in a panel with four figures (a, b, c and d), where de first would be a representation of the overall mortality rate (axis Y) and its respective confidence interval for each year (axis X). The second, third and fourth figures could follow the same logic. The Table 2 is short of objectivity.

Response: Figure 1 depicts the observed mortality rates in Brazil, and not estimates. We changed the text to make this clear. Since they are not estimates, confidence intervals are not applicable in this case, and we have decided to keep the figure as presented.

8-Regarding on limitations study, the authors should explain to the reader the expected impact of the underreporting and misclassification errors on the interpretation of main manuscript results. It would also be interesting to point out the unavailability of census 2022 data for Brazilian population and the limits of the projected population by the IBGE during 2015 to December 2022.

Response: Thank you for your input. We took your recommendations and have improved the discussion of the limitations of our study. A revised section addressing these limitations reads from lines 256-260:

References

1-Fajardo S, Aerts DR, Bassanesi SL. [Accuracy of the Mortality Information System team in the specification of underlying cause of death in a State capital in southern Brazil]. Cad Saude Publica. 2009;25(10):2218-28.

2 Paula AA, Schechter M, Tuboi SH, Faulhaber JC, Luz PM, Veloso VG, et al. Continuous increase of cardiovascular diseases, diabetes, and non-HIV related cancers as causes of death in HIV-infected individuals in Brazil: an analysis of nationwide data. PLoS One. 2014;9(4):e94636.
